# Experience developing a pediatric medical chatbot in Singapore: a digital innovation for improved emergency care

**DOI:** 10.3389/fdgth.2025.1557804

**Published:** 2025-03-18

**Authors:** Choo Min, Rong Xiu Cynthia Lim, Sek Wan Tan, Sashikumar Ganapathy

**Affiliations:** ^1^ Department of Emergency Medicine, KK Women’s and Children’s Hospital, Singapore, Singapore; ^2^ Clinical Teaching Faculty, Duke-NUS Medical School, Singapore, Singapore

**Keywords:** medical chatbot, digital health, pediatric acute care, pre-hospital triage, caregiver empowerment

## Abstract

This community case study explores the lessons learnt from the development of the Urgent Paediatric Advice Line (UPAL), a medical chatbot designed to address key challenges in pediatric healthcare, including emergency department (ED) overcrowding, health-seeking behavior, and health literacy. The chatbot was developed by pediatric specialists in collaboration with an AI-driven technology partner to provide caregivers with timely, accurate, and accessible guidance for managing pediatric health concerns. By helping parents assess the severity of their child's symptoms and navigate appropriate care pathways, UPAL aims to reduce unnecessary ED visits and improve health literacy. The development process employed an iterative, user-centered approach to refine the algorithm and enhance the user experience, with key challenges including balancing clinical reliability with user empathy. By offering evidence-based advice tailored to individual symptoms, UPAL empowers caregivers to make more informed decisions about their child's care. This case study highlights the potential of digital health solutions to empower caregivers, improve patient engagement, and increase healthcare access, particularly in pediatric settings. The study underscores the lessons for the field—namely the importance of interdisciplinary collaboration, continuous iterative development, patient-centered design, and active stakeholder engagement in creating effective digital health tools. Looking forward, future developments will include the incorporation of generative AI to provide more humanistic and personalized responses, as well as the creation of a post-discharge outreach module to provide proactive post-discharge support to caregivers, further enhancing healthcare delivery in a rapidly evolving digital landscape.

## 1 Introduction

The digital revolution, accelerated by the necessity of the COVID-19 pandemic, has significantly transformed the healthcare landscape. Telemedicine, which enables the remote delivery of medical advice and facilitates symptom triaging, has emerged as a vital tool in reducing unnecessary visits to healthcare facilities, particularly emergency departments (EDs). Traditionally, this function was managed through nurse-operated telephone triage lines, but with advancements in digital health, solutions like medical chatbots are now playing a pivotal role in optimizing healthcare resources and improving patient care.

This community case study explores the development and implementation of a pediatric medical triage chatbot designed to address several interconnected challenges: emergency department overcrowding, health-seeking behaviors, and health literacy, all while delivering patient-centered care. The primary aim of this digital solution is to empower caregivers, reduce unnecessary ED visits, and enhance access to appropriate care for children in need.

The chatbot is particularly valuable in the pediatric context for several reasons:

1. Target Population: It is specifically designed for parents of young children, a group that tends to be digitally savvy and more open to adopting new technologies.
2. ED Overcrowding: Pediatric EDs are often overwhelmed by patients with mild conditions, driven in part by parents’ heightened anxiety when their children are unwell.
3. Parental Uncertainty: Many parents, especially those with limited experience in managing their child's health, face uncertainty about when and how to seek medical care, which can lead to unnecessary ED visits.

By providing real-time, evidence-based guidance, the chatbot helps parents make informed decisions about their child's health, potentially reducing unnecessary ED visits while ensuring timely and appropriate care.

## 2 Background and rationale

### 2.1 ED crowding

ED crowding has been and continues to be a pervasive pressing issue in healthcare systems worldwide, including Singapore. Defined as “a situation in which the identified need for emergency services outstrips available resources in the ED” (1), overcrowding can have wide-reaching negative impacts, including prolonged wait times, delayed treatment, reduced quality of care, increased risk of medical errors, healthcare worker burnout, and limited hospital capacity to respond to disasters or sudden surges in disease outbreaks (1–4). This complex, multifaceted issue arises from various factors related to *input*, *throughput*, and *output* within the ED system. While all these factors contribute to ED congestion, this study focuses primarily on addressing the issue of *input*—the number of patients seeking emergency care in the first place.

*Input* is often driven by the overuse of ED services for non-urgent conditions, which further strains healthcare resources. A study conducted on an adult ED in Singapore found that children under 10 years old were the most likely to make inappropriate ED visits [odds ratio (OR) 12.89] compared to other age groups (5). Factors contributing to these inappropriate visits include caregivers overestimating the severity or urgency of their child's illness, a desire for reassurance about the seriousness of their child's condition, mistrust in primary care providers (PCPs) to manage pediatric issues, limited access to after-hours primary care, convenience of having investigations done at the same location, and financial considerations (such as insurance coverage or financial assistance) (6, 7).

To reduce *input*, efforts have focused on improving the accessibility of primary care services, offering better access to appropriate healthcare guidance, and introducing alternative care options. These include increasing access to after-hours primary care, establishing nurse triage lines, and expanding telemedicine services. Nurse triage lines can help caregivers determine whether their child's symptoms require emergency care or can be managed at home or by a PCP (8). However, nurse triage lines are resource-intensive, requiring significant staffing and operational costs. Many of the common queries that caregivers have can be effectively automated, making chatbots a potential solution. By leveraging chatbots to address routine concerns, healthcare systems can reduce the strain on live triage lines, allowing human resources to focus on more complex cases, while still providing timely and accurate information to caregivers.

### 2.2 Health-seeking behavior

Health-seeking behavior refers to the actions taken by individuals to seek care for perceived health issues, influenced by a range of psychological, social, and economic factors. In pediatric care, the behavior of parents or caregivers is particularly crucial, as they are responsible for making healthcare decisions on behalf of their children. Studies have shown that parents often exhibit health-seeking behaviors that are shaped by a combination of symptom severity, perceived urgency, and the availability of healthcare resources (9). When children are ill or injured, caregivers may experience heightened anxiety, which can influence their decision to either overestimate or underestimate the need for emergency care.

For many caregivers, particularly those with lower access to healthcare resources or lacking guidance on appropriate care pathways, the default action is often to seek care at the emergency department, even for non-urgent concerns (10). This is particularly problematic in the context of pediatric healthcare, where many conditions are self-limiting or can be managed effectively at home or through telemedicine. A lack of understanding about when to seek emergency care can lead to unnecessary ED visits, contributing further to overcrowding.

### 2.3 Health literacy

Health literacy, defined as the ability to obtain, process, and understand basic health information to make informed decisions (11), is crucial for effective healthcare management. However, research highlights a significant gap between general literacy skills and health literacy. For example, 36% of U.S. adults were found to have basic or below-basic health literacy skills (12), which can impede their ability to comprehend and act on health information. In pediatric care, this challenge is particularly pronounced, as caregivers must accurately assess their child's symptoms and decide when to seek medical advice. Many parents struggle with medical terminology, identifying warning signs, and navigating healthcare systems, leading to both unnecessary emergency department visits and delays in seeking care for more serious conditions (13, 14).

The vast amount of health information available online presents both opportunities and challenges. While reputable websites, online forums, and social media platforms have revolutionized the way individuals seek health information, they also present issues of reliability and accuracy. Individuals with low health literacy may struggle to discern credible sources from misleading or false information, which can lead to poor decision-making and negative health outcomes (15). As a result, there is a growing need for reliable, accessible, and evidence-based health resources that can guide caregivers in making informed decisions.

Digital health solutions, such as pediatric triage chatbots, offer a promising way to address these challenges. By providing easily accessible, understandable, and evidence-based information, these chatbots can help bridge the health literacy gap. Through personalized symptom assessments and clear guidance on care options, they can assist caregivers in making more informed decisions, ultimately improving health outcomes and reducing unnecessary healthcare utilization (16–19).

## 3 Program description: UPAL

This project was developed by a medical team from the Children's Emergency at KK Women's and Children's Hospital (KKH) comprising of doctors and nurses, in collaboration with KeyReply, a local vendor specializing in Artificial Intelligence (AI) for healthcare. Their proficiency in AI and chatbot technology, combined with our medical expertise in pediatric emergency care, formed a synergistic partnership for this initiative. When UPAL was launched in June 2019, it was one of the first medical chatbots for the public in Singapore. To date, it still stands as the only pediatric medical chatbot in Singapore.

**Figure 1:**
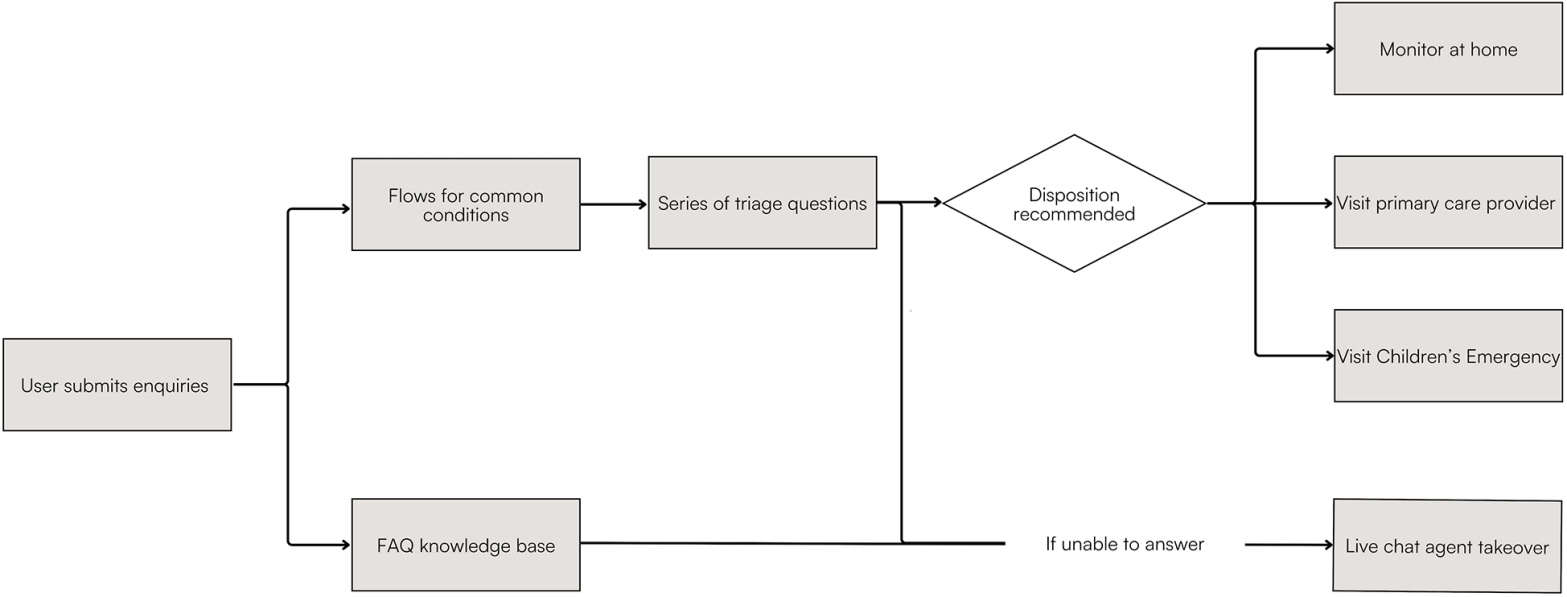
Flowchart illustrating UPAL's overall algorithm.

UPAL uses a web-based, conversational chatbot interface to answer user's queries and triage patients to different recommended levels of care. Based on the most common presenting symptoms and user queries, customized pathways were developed by the clinical team, in alignment with local clinical practice guidelines. These pathways included algorithms for conditions such as fever, cough, vomiting, and diarrhea. As illustrated in Figure 1—if a user query falls into one of the established pathways, a series of triage questions will be asked to establish the severity of illness, before recommending for the child to either be monitored at home, visit primary care providers, or the Children's Emergency (CE). If user query is regarding other topics, the chatbot will try to draw answers from a Frequently Asked Questions (FAQ) knowledge base. If at any point the chatbot is unable to answer, user will be directed to speak to a live chat agent. Live chat is manned by a CE doctor or nurse from 8am to 11pm daily.

**Figure 2:**
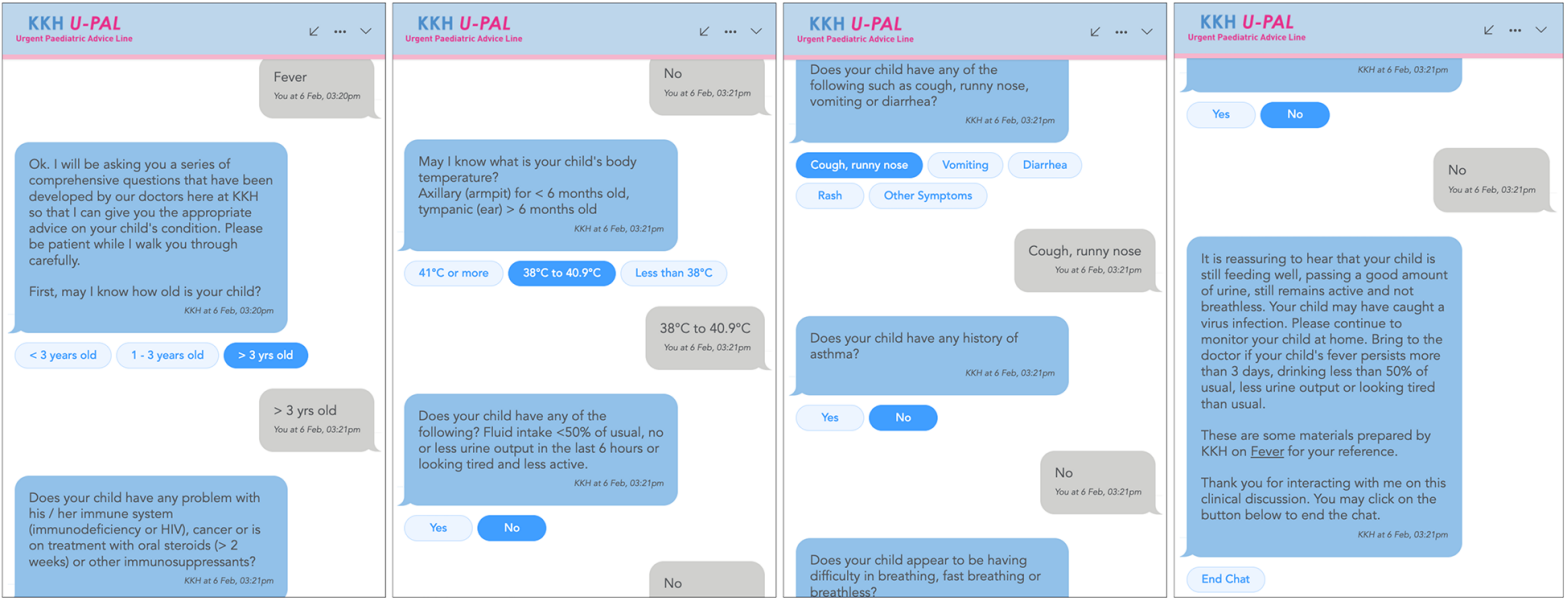
Example of a user chat interaction with UPAL.

An example illustrating a user chat interaction has been provided in Figure 2. Users did not have to register to utilize the chatbot, and patient identification details (such as identification numbers) were not collected.

On the technical end, UPAL is a rule-based chatbot that utilizes Natural Language Processing (NLP) to attempt to understand the user intent and context, before replying with predefined responses. Training the chatbot to understand user intent and context took rigorous effort, especially when there were multiple chief complaints in a single message, and with the widespread use of Singlish (a mixture of English, Mandarin, Malay, dialects) in Singapore (20).

## 4 Methods

As this is a descriptive write-up, data was collected from the operational end of the chatbot—this included information on number of chat sessions, number of users, and recommended disposition. User feedback was collected at the end of each chat session by asking users to rate their experience on a 5-point Likert scale, and to provide further feedback via free text.

## 5 Results

### 5.1 Chatbot usage

**Figure 3:**
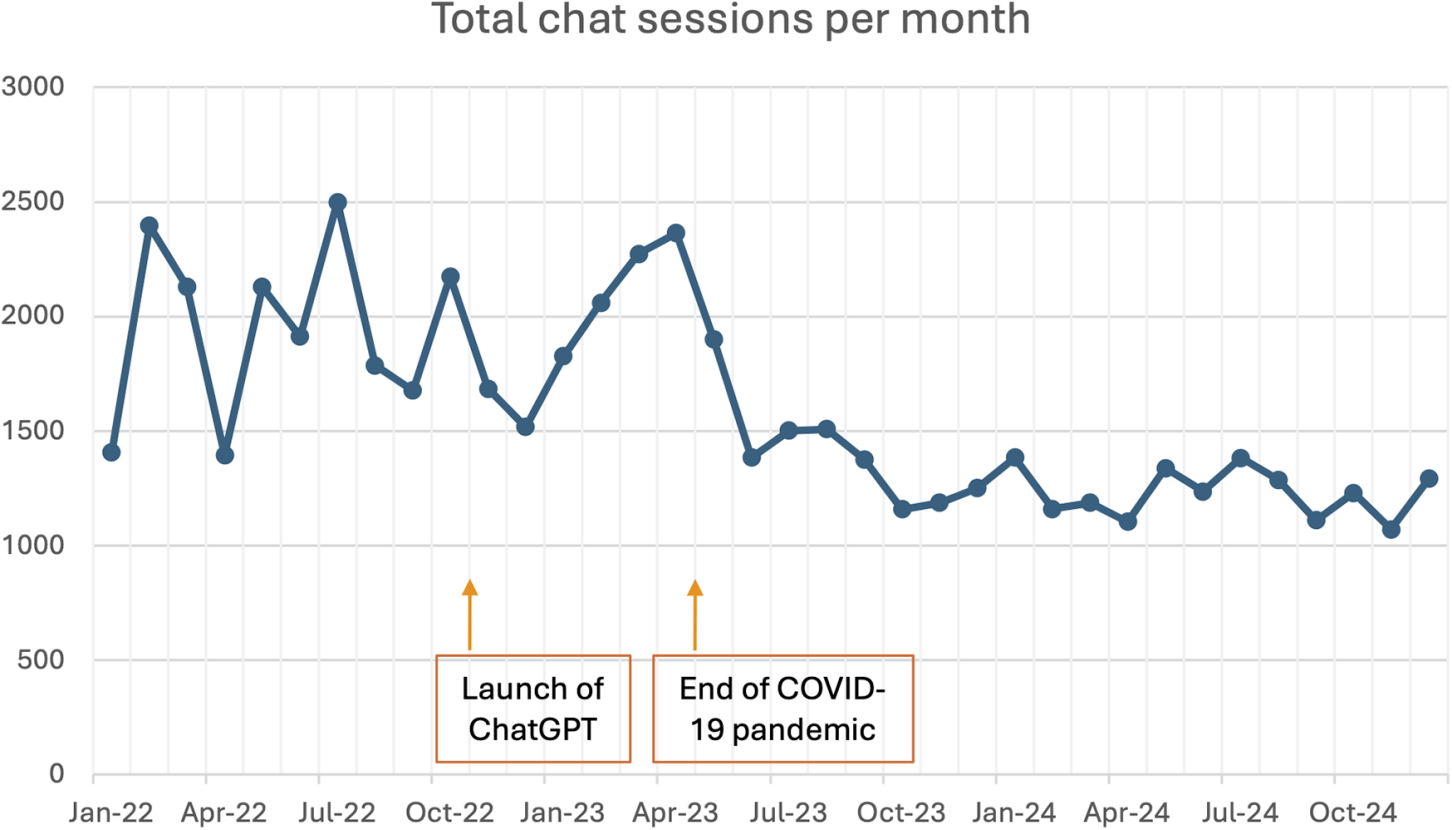
Trend of monthly volume of chats from Jan 2022 to Dec 2024.

Over the course of January 2022 to December 2024, there were a total of 57,302 chat sessions recorded. Figure 3 shows the trend in the number of monthly chat sessions, with a noted marked decline since mid 2023.

On the other hand, throughout the deployment of UPAL, the monthly percentage of repeat users (as compared to total users) remained consistent at 44%, indicating a steady base of proponents who found value and continued to engage with the chatbot.

### 5.2 Recommended disposition

Of the total 57,302 chat sessions, 14,289 sessions had completion of flow with dispositions recommended. As can be seen from Table 1, a vast majority of users sought guidance for minor health concerns, with 90.5% advised to continue monitoring at home. A further 7.7% were advised to seek medical attention from primary care providers, and only 1.8% were advised to visit the CE. This highlights the chatbot's ability to address common, low-risk pediatric conditions effectively.

**Table 1:** Breakdown of dispositions recommended for chats with completed flow (*N* = 14,289).

Disposition recommended	Count (%)
Monitor at home	12,981 (90.5)
Visit primary care provider	1,042 (7.7)
Visit children's emergency (CE)	266 (1.8)
Flows not resulting in CE	14,023 (98)

### 5.3 User feedback

Short questions soliciting quantitative and qualitative user feedback were placed at the end of each interaction with the chatbot. Although response rate was low, the feedback offered valuable insights into areas for improvement and user perceptions of the chatbot's performance.

Table 2 shows a summary of the quantitative feedback. User experience rating remained consistent over the years, with a clear higher rating for live chat sessions. For qualitative feedback, a large majority (80%) declined to give comments, leaving the chat once their needs were met. 9,455 (16.5%) gave neutral comments—these included answers with little meaningful context (such as “thank you”, “don't take over us”), and requests for more features and specific topics. Of the remaining minority, 974 (1.7%) expressed positive comments and 1,031 (1.8%) provided negative feedback.

**Table 2:** User experience rating based on 5-point likert scale.

	2022	2023	2024
Sessions completed entirely by chatbot			
Number of chat sessions	21,808	19,057	14,185
Number of survey respondents (%)	1,636 (7)	1,325 (7)	924 (6)
User experience rating^a^ (Mean ± SD)	3.37 ± 0.16	3.43 ± 0.28	3.31 ± 0.18
Sessions requiring escalation to live chat			
Number of live chat sessions	907	741	604
Number of survey respondents (%)	190 (21)	127 (17)	116 (19)
User experience rating^a^ (Mean ± SD)	4.26 ± 0.46	4.12 ± 0.55	4.29 ± 0.43

^a^ 1—strongly disagree, to 5—strongly agree.

**Figure 4:**
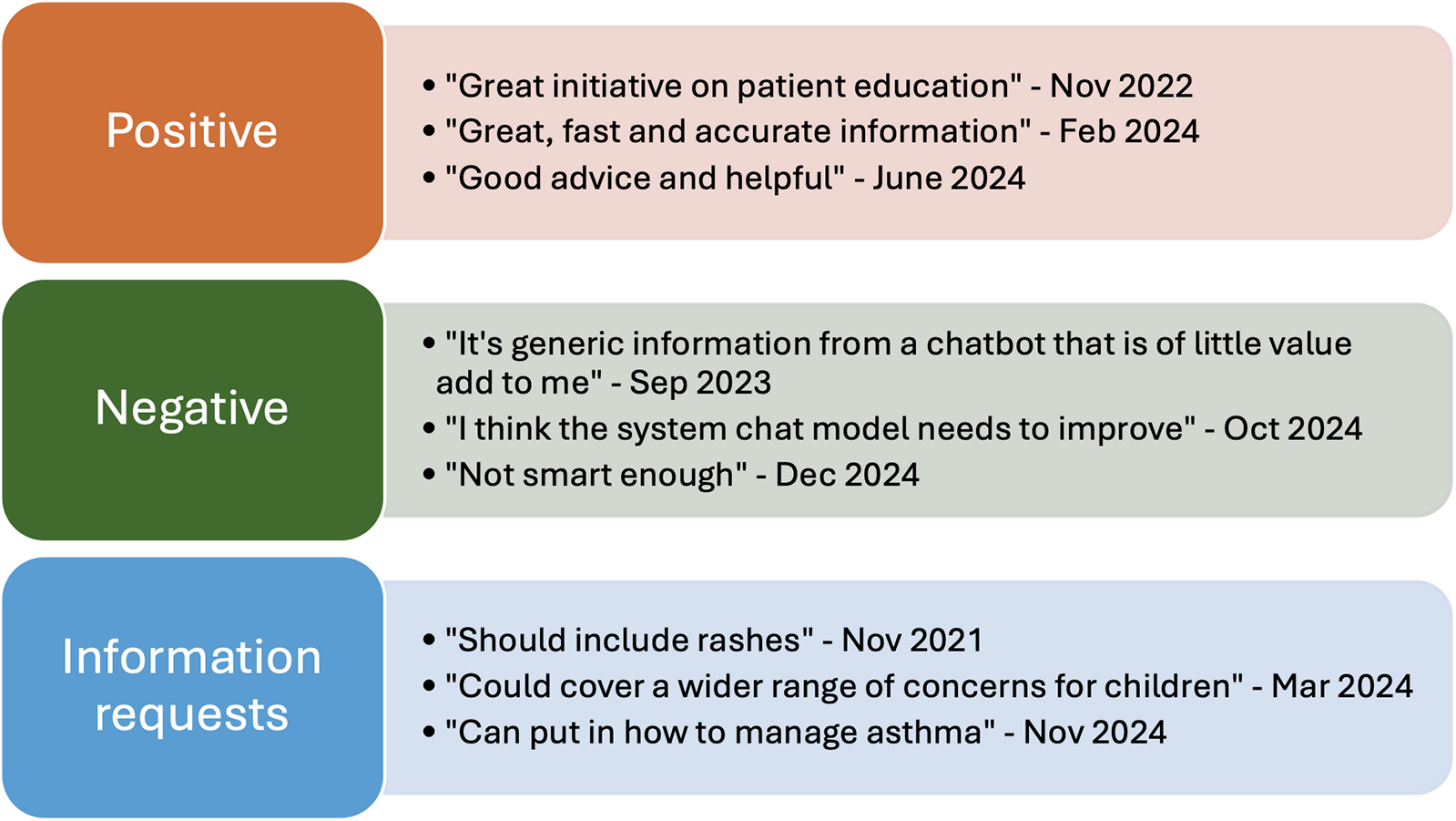
Qualitative feedback from users.

From the examples in Figure 4, positive feedback highlighted user satisfaction with the chatbot's ability to provide fast and accurate information, as well as its role in patient education. Negative comments pointed to limitations in the chatbot's functionality, with some users feeling the information was too generic or that the system needed further improvement. These suggestions indicate a desire for more personalized, comprehensive, and adaptable responses, and emphasizes the need for continuous refinement of the chatbot's capabilities.

## 6 Discussion

We postulate that the marked drop in chatbot usage since mid 2023 was attributed to multiple reasons. First and most evidently, the end of the COVID-19 pandemic in May 2023 led to a reduced fear of visiting healthcare institutions. As public anxiety decreased, caregivers reverted to their usual behavior of seeking physical medical reviews before attempting self-help through the chatbot, even for minor ailments in their children. Secondly, as users acquired more knowledge and were directed to trusted resources, the need for frequent chatbot use diminished. Thirdly, the decrease in marketing efforts due to the cessation of initial funding resulted in reduced public awareness, which hindered the chatbot's reach, especially among new parents who could benefit from the service. Lastly, the advent of ChatGPT in November 2022 set new expectations for chatbot interactions. With these more advanced systems becoming more ubiquitous, users began to expect a higher level of sophistication and personalization from chatbots, leading to some dissatisfaction with the information delivery and interaction quality in UPAL.

There were also several limitations in tracking concrete outcomes to evaluate the overall impact of UPAL. The first limitation lies in the multitude of factors affecting ED attendance, such as surges in infectious diseases, festive seasons, and the coincident introduction of other healthcare initiatives to reduce ED attendance. As a result, it was not possible to attribute reductions in ED attendance or waiting times solely to the introduction of UPAL, making it difficult to measure the chatbot's direct impact on ED traffic. Moreover, the system was designed not to collect patient identifiers such as national identification numbers, which helped mitigate privacy risks but also made it impossible to match user interactions with their medical records and track whether users followed through with the recommended dispositions.

The main objective of UPAL was to automate responses to common medical inquiries to better utilize healthcare human resources, reduce unnecessary emergency department (ED) attendances, and provide timely guidance to caregivers seeking advice for their children's health. The chatbot, developed with the aim of streamlining healthcare access, was particularly useful during the COVID-19 pandemic, when the fear of contracting the virus in healthcare settings prompted many patients to avoid physical consultations. Feedback from users highlighted the chatbot's value, especially during this period, as it offered an alternative means of obtaining medical advice without the need to visit a healthcare facility. However, like any technological innovation, the adoption and sustainability of UPAL required continuous effort, for which the learning points are expounded below.

## 7 Learning points

### 7.1 Build trust and credibility with target users

Trust is a fundamental factor in the successful adoption of medical chatbots, especially when it comes to health-related decisions. For caregivers to feel confident using a pediatric medical chatbot, it is essential that the technology is perceived as reliable, accurate, and backed by trusted healthcare institutions. A critical component of building trust was ensuring that our chatbot's triage algorithm was medically sound. The medical team had to define criteria for symptom severity, risk factors, and guidelines for when to advise parents to seek immediate medical care. There was also continuous clinical oversight throughout every stage of the chatbot's development.

### 7.2 Importance of interdisciplinary collaboration

A significant learning point was the importance of collaboration between experts from both the healthcare and technology sectors. Even with the best medical knowledge, if the chatbot's user experience was suboptimal or its value was unclear, users would be reluctant to engage with it. The project demonstrated the power of interdisciplinary collaboration, where healthcare professionals worked closely with AI experts to translate medical knowledge into a functional chatbot algorithm. The medical team ensured clinical accuracy, while the technical team contributed their expertise in machine learning to enhance the chatbot's ability to analyze symptoms, age, and medical history in real time, providing relevant advice. An ongoing, transparent feedback loop between the medical and technical teams was crucial to maintaining the chatbot's clinical precision and ensuring it continued to deliver value to users.

### 7.3 Continued iterative development is crucial

A key learning point from the development process was the critical need for ongoing iterative development to expand and update the knowledge base, and refinement of the triage algorithms to maintain the chatbot's effectiveness. To ensure safety and consistency, the chatbot underwent multiple rounds of testing by the clinical team with simulated triage scenarios before launching to the public. Post launch, chat logs and user feedback were made available to the clinical team via a dashboard, to facilitate relay of real-time data and analysis to identify areas for improvement and prioritize updates. The clinical and technical team continued to meet regularly to work on content updates in the background, without affecting the running of the chatbot in the foreground.

Such updates included gradual addition of triage algorithms on rashes, COVID-19, trauma and testicular torsion; and FAQ knowledge content such as information on infant feeding and crying. This was in response to user queries and feedback, to ensure that UPAL stays relevant and meets the evolving needs of end-users through continued small cycles of change.

### 7.4 Understanding and anticipating user needs: a patient-centered design

Another important lesson was the necessity of designing the chatbot with a patient-centered approach. Beyond simply offering a functional tool, the chatbot needed to add value to users’ lives, particularly in a pediatric setting where caregivers are often under stress, feeling anxious or uncertain about their child's health. A critical element of this was the chatbot's ability to communicate in a clear, concise, and empathetic manner.

However, during the early stages, the chatbot's responses lacked the human touch that users expected. The algorithms driving the chatbot were mechanical, providing functional responses but failing to convey warmth and empathy. As reported in the results section, feedback for live chat remained consistently higher than that for sessions completed entirely by chatbot algorithms. There was also a group of users familiar with the chatbot who knew how to navigate the system effectively, escalating their inquiries to a live chat frequently. This revealed that users were seeking more humanistic, personalized interactions. This realization led to an emphasis on improving the chatbot's emotional intelligence, by intentionally placing empathetic phrases at multiple points along the triage algorithm.

### 7.5 Engage stakeholders early and continuously

Engaging stakeholders early and continuously was essential to the success of the pediatric medical chatbot. While the primary users of the chatbot were caregivers seeking advice about their children's health, several other groups played pivotal roles in its development and ongoing success. These included the medical team responsible for creating the algorithms, the nurses managing the live chats, and hospital leadership, who provided oversight and support. By fostering an environment of mutual respect and recognition, the project gained the trust and commitment of everyone involved, which helped sustain enthusiasm and motivation over time.

One of the initial challenges faced by the live chat nurses was their lack of confidence in handling anticipatory care questions. As these were nurses trained primarily in emergency care, they excelled in addressing urgent situations but felt less equipped to handle inquiries related to preventive measures and long-term health strategies. Offering ongoing training helped alleviate some of this discomfort and build their confidence in responding to such queries. Additionally, the initial live chat system presented logistical challenges. Nurses had to use desktop computers to respond, which led to slower response times and decreased flexibility. The system was thus upgraded to allow nurses to respond via mobile phones, which made it easier to manage live chat inquiries on the go, and ensure that nurses did not feel overburdened by the system. Recognizing and addressing their concerns allowed the team to provide a more effective and supportive role, helping to maintain engagement and reduce feelings of burnout.

The other important stakeholder was hospital leadership, to secure buy-in for the long-term sustainability of the pediatric medical chatbot. When initial funding for the chatbot's development ran out, there was a noticeable decline in marketing efforts, leading to reduced public awareness and decreased the reach of the chatbot, particularly among new parents. With concerns mounting about whether the chatbot would continue to operate without sustained financial support, it became evident that continued hospital leadership backing was crucial to keep the system viable. Buy-in from hospital leadership is vital not only for initial development but also for ongoing sustainability, as their support helps ensure the project aligns with broader organizational goals and is sufficiently resourced.

## 8 Future work

Moving forward, several key enhancements are in the pipeline to improve UPAL's effectiveness, user experience, and overall impact on healthcare delivery.

### 8.1 Incorporation of generative AI

The rapid advancements in AI, particularly with the introduction of generative models like ChatGPT in 2022, have significantly enhanced the capabilities of chatbots. One of the most promising developments currently planned for UPAL is the integration of generative AI, which would enable it to engage in more natural, contextually aware, and human-like conversations. Unlike traditional rule-based systems that rely on predefined responses, generative AI can understand and process complex, nuanced user inputs, allowing it to generate replies that feel more personalized and empathetic. This improvement would elevate the overall quality of the chatbot's interactions, making them more relatable and emotionally supportive for caregivers.

Additionally, incorporating generative AI would vastly improve the chatbot's scalability. It would enable the system to handle a wider array of inquiries, from general pediatric concerns to more complex, less common issues, all while adapting to a broader spectrum of user inputs. The chatbot could learn and evolve over time, responding more effectively to diverse linguistic styles and variations, including colloquial or culturally specific language. Moreover, it could tailor its communication style to meet the emotional needs and preferences of individual users, providing a more customized experience. Overall, the integration of generative AI promises to make the chatbot a more versatile, efficient, and empathetic tool for caregivers seeking support in pediatric healthcare.

### 8.2 Incorporation of post-discharge materials

Another key area for future work is the integration of post-discharge materials, such as discharge advice, pamphlets, and instructional videos, directly into the chatbot's interface. After a caregiver has visited the Children's Emergency or used the chatbot for symptom assessment, we plan to offer relevant post-care resources through UPAL to ensure ongoing support. These materials would provide caregivers with the information they need to care for their child at home, reinforcing advice on follow-up care, managing common symptoms, and knowing when to seek further medical attention.

### 8.3 Outreach module for proactive post-discharge support

To further enhance the chatbot's role in pediatric care, an outreach module will be developed to provide proactive post-discharge support. This feature will allow the chatbot to initiate regular check-ins with caregivers after their child has been assessed or discharged. These check-ins will include reminders about follow-up appointments, advice on when to return for a medical review, or even just a wellness check to monitor the child's recovery. This proactive support will potentially help prevent complications, improve health outcomes, and reduce the likelihood of unnecessary readmissions or ED visits.

Together, these enhancements will not only improve the chatbot's functionality and scalability but will also deepen its ability to provide comprehensive, patient-centered care throughout the entire healthcare experience.

## Data Availability

The original contributions presented in the study are included in the article/Supplementary Material, further inquiries can be directed to the corresponding author.
